# The Modified THP-1 Activation Assay for the *In Vitro* Identification of Drug-Inducing Systemic Hypersensitivity

**DOI:** 10.3389/ftox.2022.814050

**Published:** 2022-03-03

**Authors:** Martina Iulini, Ambra Maddalon, Valentina Galbiati, Emanuela Corsini

**Affiliations:** Laboratory of Toxicology, Department of Pharmacological and Biomolecular Sciences, Università Degli Studi di Milano, Milan, Italy

**Keywords:** *in vitro* method, NAM, drug hypersensitivity, THP-1 activation assay, surface markers, IL-8

## Abstract

The development of new low molecular weight drugs has many chances of failure and is an expensive process. Currently, there are no screening methods and/or models to assess the hazard of hypersensitivity reactions to drugs (DHRs) in the preclinical phase. DHRs represent 6–15% of adverse drug reactions. Although rare, DHRs represent a serious health problem for predisposed individuals, resulting, in some cases, in life-threatening pathologies. To date, there are no *in vitro* or *in vivo* sensitive models able to predict the sensitizing potential of drugs in the preclinical tests, and these reactions are highlighted only after the drug has been placed on the market, affecting both population and public health. This article describes a novel approach methodology for the study of the sensitizing potential of drugs based on the use of the human promyelocytic cell line THP-1 as a surrogate for dendritic cells. The method is based on the upregulation of specific surface markers (CD86 and CD54) and on the production of IL-8. In our experience, the THP-1 activation assay allowed the correct identification of drugs known to induce systemic hypersensitivity in humans, including the one associated with specific HLAs. This method may help to discover possible systemic hypersensitivity reactions early in the preclinical phase of drug development.

## 1 Introduction

Adverse drug reactions are due to different mechanisms and give rise to different clinical pictures. As proposed by Rawlins and Thompson (Rawlins and Thompson, 1977), they can be classified into two types. Type A reactions represent 85–90% of all reactions and are usually dose-dependent, related to the known pharmacological actions of the drug, and occur in otherwise health subjects. Type B reactions, instead, account for approximately 10–15% of all the reactions, are generally dose-independent, unrelated to the pharmacological actions of the drugs, occur only in susceptible subjects, and involve immune-mediated reactions or drug allergies (Hausmann et al., 2012). The majority of type B reactions are drug hypersensitivity reactions (DHRs); immune-mediated adverse reactions are the most frequent immunotoxic effects of drugs. DHRs include immune-mediated and non–immune-mediated reactions that are linked to the intrinsic proprieties of drug molecules and the genetic predisposition of the patient. The most common allergic reactions occur in the skin and are observed in approximately 2–3% of hospitalized patients (Pichler, 2004). DHRs can also be quite serious, e.g., life-threatening, and they represent a problem in drug development because they are usually only apparent after the drug has been put onto the market due to the lack of predictive experimental models in the preclinical phase. The problem of non-predictability of immunologically based hypersensitivity reactions is related to the lack of appropriate experimental models rather than to the lack of understanding of the adverse phenomenon (Galbiati et al., 2016). Generally, animals are good predictors of human response to drugs; however, for some categories of toxicity, including hypersensitivity reactions, animal models are not considered completely adequate to predict the potential human toxicity because young and healthy animals are tested (Ogese et al., 2017). This calls for the need to develop *in vitro* assays to detect the sensitizing potential of drugs in the preclinical phase.

DHRs are based on three distinct mechanisms: the hapten mechanism, the pharmacological-interaction (p-i) concept, and the pseudo-allergic mechanism. The first one is based on the covalent binding of drugs to proteins, with the development of antigens from which an immune response can develop (which can be both humoral and cellular) (Pichler, 2003). The second involves the interaction of the drug with the immune receptor such as the human leukocyte antigens (HLA) or T-cell receptor, resulting in direct stimulation of T cells (Pichler, 2002). Some of these p-i stimulations only occur in carriers of certain HLA alleles and can cause clinically severe reactions (Zanni et al., 1998). The third mechanism is represented by the interactions between the drugs and the receptors or enzymes of inflammatory cells, which can lead to their direct activation or to an increase in the levels of inflammatory products (McNeil et al., 2015). This classification is based on drugs’ action and explains differences in sensitization, unusual clinical symptoms, dependence on drug concentrations, immunological and pharmacological predictability, and cross-reactivity in DHRs (Pichler, 2019).

The cells involved and mediators released during the different phases of hypersensitivity reactions can be assessed using different *in vitro* diagnostic tests. The methods used for the diagnosis of DHRs depend on the mechanism involved and the kinetic of the reaction. They can be divided in tests able to identify the drugs at the resolution of the reaction or allow determining the individual risk of DHRs before drug administration. *In vitro* methods, such as the myeloid U937 skin sensitization test, the human cell line activation test (h-CLAT), and the THP-1 activation assay, may be used in the preclinical phase of drug development for hazard identification of the potential to induce hypersensitivity reactions (Galbiati et al., 2016). All three methods mentioned are based on the key mechanistic events underlying the awareness process described in the OECD report on “The Adverse Outcome Pathway (AOP) for Skin Sensitization Initiated by Covalent Binding to Proteins” (OECD, 2012). For all the *in vitro* methods mentioned, the hypothesis is that traditional drugs or drug metabolites have low molecular weights (< 1,000 Da), and as such, they are too small to be “seen” by T cells, and for these reasons, they are unable to spontaneously give an immune reaction. However, similar to chemical sensitizers, they can act as haptens by stably binding to carrier proteins and forming complete high molecular weight immunogenic compounds. Dendritic cells (DCs) subsequently process this hapten and differentiate into a mature phenotype, characterized by the high expression of costimulatory molecules (CD80, CD86, and CD40), adhesion molecules (CD2, CD11a, CD54, and CD58), and release of cytokines (IL-1β, IL-18, and IL-8) (Quah and O’Neill, 2005). After stimulation, a clone of T cells is produced capable of reacting to the antigen and therefore causing DHRs (Martin, 2012).

Starting from the evidence that sensitizing drugs share the same mode of action with chemical sensitizers, we proposed to adapt the THP-1 activation assay, developed for skin and respiratory sensitizers, also for the identification of drugs that may be associated with *in vivo* drug hypersensitivity reactions. We established experimental conditions and markers to correctly identify drugs associated with hypersensitivity reactions *in vivo* using an *in vitro* approach, based on THP-1 cells and the production of IL-8 and the expressions of CD86 and CD54 (Corti et al., 2015; Iulini et al., 2020). The method proposes the study of DC activation as a simple and reproducible tool to predict the potential hazard of drugs to induce DHRs. The protocol could be also useful for testing metabolites or drugs for which metabolism is needed. The THP-1 activation assay is a test that can be easily integrated into drug development for the preliminary identification of drug-induced immune-mediated hypersensitivity reaction risks.

The THP-1 activation assay is an alternative *in vitro* test that allows evaluating the allergenic potential of low molecular weight chemicals based on the molecular mechanisms underlying skin and systemic sensitization. It was developed during the European project SENS-IT-IV to exploit the ability of the human THP-1 cell line to identify contact and respiratory allergens. The method was based on the evaluation of the production of IL-8 and the expressions of CD86 and CD54, for the identification of allergic compounds (Mitjans et al., 2008; Mitjans et al., 2010). IL-8 is a chemotactic peptide for T lymphocytes and neutrophils, shown to be a useful biomarker to discriminate sensitizers from non-sensitizers (Toebak et al., 2006; Kimura et al., 2015). It was also demonstrated that the release of IL-8 could provide indication on the potency of allergens as it shows a good correlation with the LLNA EC3 values (Mitjans et al., 2010). In addition to the investigation of IL-8 production, the THP-1 activation assay includes the analysis of the CD86 expression alone and/or in combination with the CD54 expression for the identification of chemical sensitizers. CD54 and CD86 are also the parameters investigated in the h-CLAT, a validated *in vitro* method (OECD TG442 E). In our experience, the evaluation of CD54 and CD86 expressions was less sensitive than IL-8 production as they failed to correctly identify approximately 30% of the tested compounds (Mitjans et al., 2008). This last observation is also in agreement with the observation found by Toebak et al. (2006); for this reason, IL-8 was included in the evaluation of chemical sensitizers, differentiating this assay from the h-CLAT.

The modified THP-1 activation assay is a tiered approach. At first, the effects on the IL-8 release and CD86 expression after 24 h of drug treatment at increasing concentrations are evaluated. If positive (statistically significant release of IL-8 and/or an SI ≥ 1.5 for CD86 at any concentration tested), the drug is considered as a potential sensitizer. If negative, in order to exclude any drug-induced activation of THP-1 cells, the IL-8 release and CD86 expression are evaluated at 48 h (positivity means statistically significant release of IL-8 and/or an SI ≥ 1.5 for CD86 at any concentration) together with the CD54 expression at 48 h (SI ≥ 1.5) and IL-8 mRNA expression at 3 h (2^−ΔΔCt^ ≥ 3.0) (Galbiati et al., 2012; Corti et al., 2015; Iulini et al., 2020). Only if negative results were obtained in all parameters, the drug will be considered a non-sensitizer.

Drugs tested were selected based on clear *in vivo* immune-adverse reactions reported in the literature and on the commercial availability as pure drugs. Several types of drugs with different structures, mechanisms of action, therapeutic applications, and immune effects have been included. The main aim of the proposed protocol is to support the evidence of *in vivo* DHRs using an *in vitro* approach. The drugs tested include the following: clonidine, ofloxacin, procainamide, streptozotocin, and sulfamethoxazole that were associated with a relatively high incidence of immune-mediated hypersensitivity reactions (Weaver et al., 2005); methyl salicylate and probenecid that can cause irritant or allergic contact dermatitis and anaphylactic reactions (Corti et al., 2015); and abacavir, carbamazepine, clozapine (Iulini et al., 2020), allopurinol, and flucloxacillin (data not already published) that were associated with DHRs, for which a correlation with specific HLAs was established. With reference to these last three drugs, the analysis of another cell surface marker, HLA-DR, necessary for the presentation of the antigen and the activation of T cells, was added to our tiered approach (Iulini et al., 2020). The HLA-DR expression is analyzed after 72 h of drug treatment. The measurement of the HLA-DR expression provides further indication of the ability of a drug to activate DCs and consequently T cells. Finally, sulfamethoxazole and procainamide were analyzed to determine the possible involvement of the metabolism.

**FIGURE 1:**
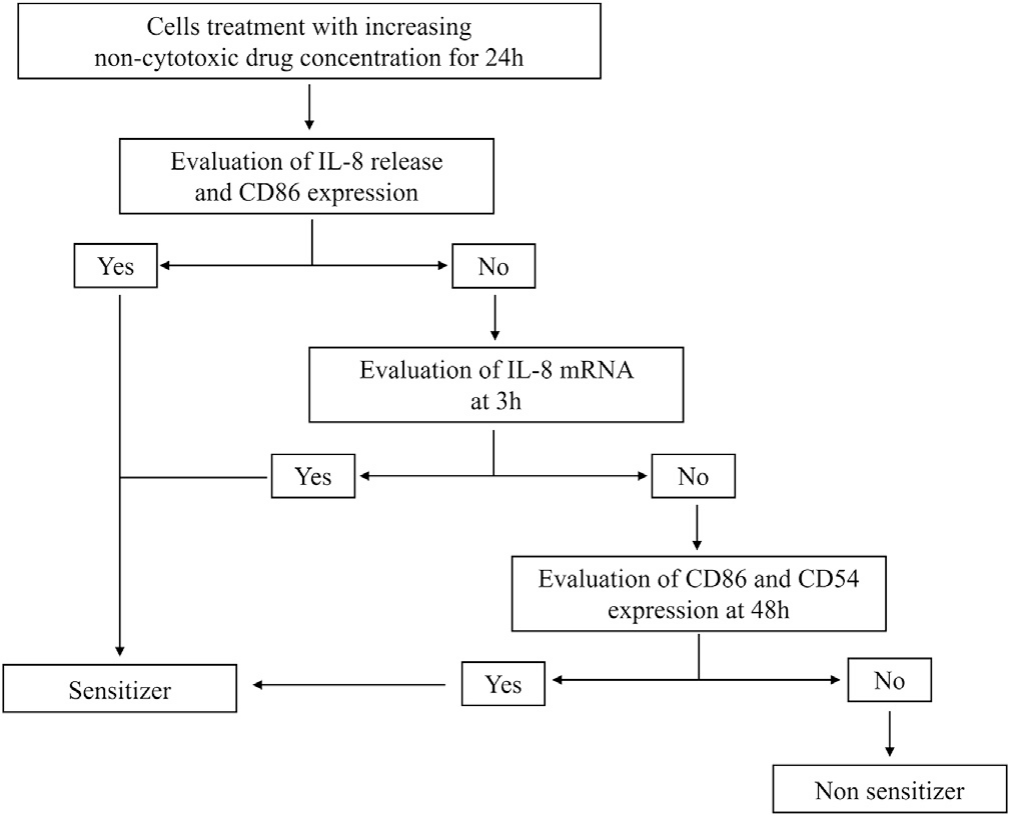
Modified THP-1 activation assay for the hazard identification of potential drug sensitizers. Graphical representation of the modified THP-1 activation assay protocol to derive the potential about sensitizing or non-sensitizing information of drugs.

Data generated demonstrate that the aforementioned novel approach methodologies (NAM) are a valid tool to predict the sensitizing potential of drugs as first screening. The THP-1 activation assay is intended to be part of a battery of tests for a more in-depth investigation. The tiered approach is shown in Figure 1.

## 2 Materials and Equipment

The materials and equipment used are as follows. Brands and CAS numbers are reported in (Tables 1 and Table 2).

**TABLE 1:** Products.

Products	Company	Catalog no
0.2 ml thin-walled tubes	BIOplastics BV	K77311
1.5 ml micro test tubes	Eppendorf	Z606340
15 ml tubes	Corning	430,766
24-well plate	Corning	3,526
5 ml combitips	Eppendorf	0030089456
50 ml tubes	Corning	430,291
96-well flat-bottom, ELISA plates	Corning	3,369
100 mm × 200 mm Petri dishes	Corning	430,167
Adjustable micropipette	Gilson	-
Adjustable multi-step pipette	Gilson	-
Autoclavable polypropylene desiccators	Thermo Scientific Nalgene	5,309–0,250
Centrifuge	Eppendorf	5,702
Chemical fume cabinet	Pharma Works Milano	PWM-IB17
ELISA Plate reader	Molecular Device	EMax Precision Microplate Reader
Flow cytofluorimeter	ACEA Biosciences, Inc	NovoCyte 3,000
Flow cytometry tubes	Greiner Bio-One	115,101
Freezer -20 °C	Ok	OFZ44214A1
Freezer -80 °C	Angelantoni industrie	Platilab 370
Hard-Shell PCR Plates 96-well, thin wall	Bio-Rad	HSP9601
High Speed Micro-Centrifuge	SCILOGEX	D3024
Incubator, 37 °C, 5% CO_2_, 95% humidity	Thermo	3,111
Inverter microscope	Wilovert	Hund wetzlar
Laboratory balance (accuracy 0,1 mg)	Gibertini	E42
Laminar flow cabinet	Gelaire	BSB 4 A
Microseal ‘B’ seal	Bio-Rad	MSB1001
Nanodrop	GE Healthcare	NanoVue™ Plus Spectrophotometer
Neubauer chamber	Hausser Scientific	-
Plate sealer	Sigma	Z369659
Refrigerator +4 °C	Beko	25,293
Thermoblock	International PBI	-
Thermal Cycler	Bio-Rad	CFX Connect™
		Real-Time PCR
Vortex	Stuart	SA8
Waterbath	Grant	JB Aqua 5 Plus

**TABLE 2:** Reagents.

Products	Company	Catalog no
2-mercaptoethanol	Bio-Rad	161–0,710
2-propanol	Sigma	I9516
3, 3′,5,5′-Tetramethylbenzidine (TMB) liquid substrate for ELISA	Sigma	T4444
Bovine serum albumin (BSA)	Sigma	A2153
Chloroform	Sigma	366,919
DMSO	Sigma	D4540
Dulbecco’s phosphate-buffered saline (PBS)	Sigma	D8537
Ethanol absolute anhydrous	Carlo Erba reagents	414,605
FITC Mouse anti-human CD86 monoclonal antibody	BD Pharmingen	555,657
FITC Mouse IgG1, *κ* Isotype Control	BD Pharmingen	555,748
Gentamycin	Sigma	G1272
Glycerol	Fisher Bioreagents	56-81–5
Heat-inactivated fetal calf serum (FCS)	Sigma	F7513
Human IL-8 ELISA	ImmunoTools	31670089
l- glutamine	Sigma	G7513
Nuclease-free water	QIAGEN	129,117
PE Mouse anti-human CD54 monoclonal antibody	BD Pharmingen	555,511
PE Mouse IgG1, *κ* Isotype Control	BD Pharmingen	555,749
Propidium iodide solution	Sigma	P-4864
QuantiNova Reverse Transcription Kit	QIAGEN	205,411
QuantiNova SYBR Green PCR Kit	QIAGEN	208,054
QuantiTect Primer Assay Hs_CXCL8_1_SG	QIAGEN	QT00000322
QuantiTect Primer Assay Hs_RRN18S_1_SG	QIAGEN	QT00199367
RPMI 1640 culture medium	Sigma	R5886
THP-1 cells	Elabscience Biotechnology Inc	EP-CL-0233
TRI Reagent	Sigma	T9424
Trypan Blue 0.4%	Sigma	T8154

### 2.1 Laboratory Equipment

5 ml combitips^TM^.

Adjustable micropipettes 0,5–10 μl, 2–20 μl, 20–200 μl, and 200–1,000 μl

Adjustable multi-step pipette.

Autoclavable polypropylene desiccators.

Microseal “B” seals.

Neubauer chamber.

Plate sealer.

### 2.2 Laboratory Devices

Centrifuge.

Chemical fume cabinet.

*In vitro* identification of drugs.

ELISA plate reader equipped with 595 nm and 405–450 nm filters.

Flow cytofluorimeter.

Freezer −20°C.

Freezer −80°C.

High-speed microcentrifuge.

Incubator, 37°C, 5% CO2, and 95% humidity.

Inverted microscope.

Analytical balance.

Laminar flow cabinet.

Nanodrop.

Refrigerator +4°C.

Thermoblock.

Thermocycler.

Vortex.

Waterbath.

### 2.3 Plates and Petri Dishes

  24-well plates.

96-well flat-bottom, ELISA plates.

100 mm × 20 mm Petri dishes.

Hard-Shell PCR Plates (96-well, thin wall).

### 2.4 Tubes and Microtubes

  0.2 ml thin-walled tubes.

1.5 ml micro test tubes.

15 ml tubes.

50 ml tubes.

Flow cytometry tubes.

### 2.5 Cell Culture Reagents

  2-mercaptoethanol.

Dulbecco’s phosphate-buffered saline (PBS).

Gentamycin.

Glycerol.

Heat-inactivated fetal calf serum (FCS).

l- glutamine.

Penicillin/streptomycin (Pen/Strep).

RPMI 1640 culture medium.

THP-1 cells.

Trypan Blue 0.4%

### 2.5 Reagents

  2-propanol.

3, 3′,5,5′-Tetramethylbenzidine (TMB) liquid substrate for ELISA.

Bovine serum albumin (BSA).

Chloroform.

DMSO.

Ethanol absolute anhydrous.

FITC Mouse anti-human CD86 monoclonal antibody.

FITC Mouse IgG1, *κ* Isotype Control.

Human IL-8 ELISA.

Nuclease-free water.

PE Mouse anti-human CD54 monoclonal antibody.

PE Mouse IgG1, *κ* Isotype Control.

Propidium iodide solution.

QuantiNova Reverse Transcription Kit.

QuantiNova SYBR Green PCR Kit.

QuantiTect Primer Assay Hs_CXCL8_1_SG.

QuantiTect Primer Assay Hs_RRN18S_1_SG.

TRI Reagent.

### 2.6 Information About the Cell Line Procedure

Master cell and working cell bank—THP-1 cells can be obtained from Elabscience Biotechnology Inc, Cat. N. EP-CL-0233 (14,780 Memorial Drive, Suite 216, Houston, Texas 77,079—Tel. 1-240–252-7368—e-mail: orders@elabscience.com or techsupport@elabscience.com).

Freezing medium—Cells are frozen in RPMI-1640, 20% FCS, and 10% glycerol at about 2 × 10^6^ cells/vial in liquid nitrogen. The freezing medium is prepared fresh when needed.

Note: the handling of this cell line is not very different from any other cell lines. No specific recommendations applied.

### 2.7 Formulation of Solutions for Sandwich ELISA

- Washing buffer: PBS + 0.05% Tween 20
- Coating buffer: PBS
- Blocking buffer/Reagent diluent: PBS + 2% BSA
- Capture antibody: reconstitute lyophilized anti-human IL-8 capture antibody in 500 μl of PBS and vortex gently to mix.
- Concentrated standard: reconstitute lyophilized recombinant human IL-8 standard (50 ng) in 1 ml of the reagent diluent and vortex gently to mix.
- Detector Antibody: reconstitute lyophilized anti-human IL-8 detector antibody in 500 μl of blocking buffer and vortex gently to mix.

### 2.8 Formulation of Solutions for RNA Isolation

- Ethanol 75% in nuclease-free water: dilute ethanol absolute anhydrous in nuclease-free water (e.g., for 10 ml: 7.5 ml of ethanol absolute anhydrous + 2.5 ml of nuclease-free water).

## 3 Methods

### 3.1 Cell Maintenance and Culture

The human leukemia cell line THP-1 should be used to perform this protocol.

1. THP-1 cells are cultured in the RPMI-1640 medium supplemented with 2 mM l-glutamine, 100 IU/ml penicillin, 100 μg/ml streptomycin, 0.01 ng/ml of gentamycin, 2-mercaptoethanol 50 μM, and 10% fetal calf serum (FCS) (heat-inactivated by heating at 56°C for 30 min, or heat-inactivated serum is bought from the supplier).
2. Maintenance of cell culture: all culture reagents (e.g., culture medium) should be warmed to 37°C before use. Cells are cultured in 100 mm Petri dishes at the density of 0.1–0.2 × 10^6^ cells/ml and maintained at densities from 0.1 to 0.8 × 10^6^ cells/ml. The cell density should not exceed 1 × 10^6^ cells/ml. Subculturing of the cell line occurs every 3/4 days. The cell culture is maintained by splitting cells and adding a fresh medium. Depending on the cell density, 2/3 new Petri dishes are obtained from a single Petri dish with a final dilution of 1:3 in the new medium. Cells are maintained at 37°C, 5% CO_2_, 95% humidity.
  Typically, cells are passed on Monday and Friday.
3. After thawing from liquid nitrogen, cells should be used for test after 3 weeks of culture, approximately 4-5 passages. Consistent results were obtained culturing cells up to 5 months; longer periods have not been tested. Therefore, cells can be used between 3 weeks after thawing, up to 5 months after thawing. Before using the cells for testing, the response to lipopolysaccharide (LPS) should be performed. The cells will be considered ready to be used if, following dose-response treatment with LPS at 6 and 24 h, we obtain a statistically significant expression compared to the control of the analyzed markers (e.g., CD86, CD54, IL-8, and TNF-α).

### 3.2 Dilution of Test Substances

1. For solid compounds, the assumption is made that 1-g compound equals 1 ml volume. When the maximum solubility is 500 mg/ml, 500 mg compound is weighed, and 500 μl vehicle is added to obtain 500 mg/ml. In any case, the solubilities indicated by the manufacturer are to be followed. Viscous compounds should be treated as solid compounds and weighted.
2. When using liquid drugs, the drugs are weighed, and the vehicle is added until a total volume of 1 ml is reached.
3. Fresh stock solutions should be prepared prior to any experiments. Depending on solubility, the drugs are dissolved either in a culture medium, in PBS, or in DMSO, when insoluble in PBS (final in-well vehicle concentration: < 0.2% v/v DMSO). The drug solutions should never be filtered. Choice of vehicle (culture medium, PBS or DMSO): the vehicle which results in the highest maximum solubility is selected. When maximum solubility is the same for both vehicles, PBS is preferred over DMSO.

### 3.3 Assessment of Cell Viability (Range Finding Study).

Two independent experiments are performed to determine the CV75% (the concentration of the tested chemical resulting in 75% viability). Cytotoxicity is assessed by flow cytometric evaluation of propidium iodide (PI)-stained cells, following 24 and 48 h of treatment.

Day 1
*1.* *Cell count:* the cells are removed from the incubator, and under a sterile laminar flow cabinet, they are transferred into a 50 ml tube, then centrifuged at 1,200 rpm for 5′ at 25°C. The supernatant is discarded, and the pellet is resuspended in 5 ml of fresh medium. To carry out the cell count, 80 µl of sterile PBS, 10 µl of Trypan Blue 0.4%, and 10 µl of the cell suspension are added into a 1.5 ml micro test tube. It is then mixed, and 10 µl of the solution is taken for cell counting using a counting chamber (e.g., Neubauer chamber) or an equivalent cell counter.
  2. Bring the cells to 10^6^ cells/ml.
  3. For each of the drugs under consideration, fresh stock solutions are prepared. A total of five 1:2 serial dilutions (concentrations 1 to 5) are recommended. A 1.5 ml micro test tube containing the 500X of the highest concentration to be tested in the cells is prepared (Conc 1), and from this, the lowest concentrations are obtained using the 1:2 dilution ratio (Conc 2, Conc 3, Conc 4, and Conc 5 — working solutions).
  4. Treatment solutions are prepared by adding the working solution to cells. We obtained five different treatment solutions with five different concentrations. For vehicle control, PBS or DMSO is added to the culture medium (see treatment example in Table 3).
  5. 500 μl of the cell treatment solution is plated in each well. In one well plate, 500 μl of THP-1 without any treatment is plated (Unstained–Unst) (see plate configuration in Table 4). The cells are incubated for 24, 48, and 72 h at 37°C, 5% CO_2_, and 95% humidity.
Days 2, 3, and 4:
  1. After 24, 48, and 72 h, the tubes are prepared for flow cytometric analysis, following the diagram mentioned as follows (Table 5).
  2. The plate containing the treatment is checked under an optical microscope.
  3. It is mixed well, and 450 μl is taken from each well and transferred in the respective tube.
  4. It is centrifuged at 1,200 rpm for 5′ at 4°C.
  5. The supernatants are eliminated, being careful not to lose the pellet.
  6. 200 μl of PBS is added for each tube, mixed, and centrifuged again at 1,200 rpm for 5′ at 4°C.
  7. The supernatants are eliminated, being careful not to lose the pellet.
  8. The PI solution with a final concentration of PI 0.625 μg/ml is prepared. 500 μl of PI solution is added in each tube.
  9. The tubes are covered with an aluminum foil (PI is photosensitive), and the lecture is started at the flow cytometer with the acquisition channel FL-3. A total of 10′000 events (cells) are acquired. Note: the analysis of all the tubes should last for maximum 30’. If not, the analysis is not accurate.

**TABLE 3:** Treatment example.

Ctrl	2 ml of cells +4 μl PBS or DMSO	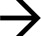 0.5 ml for well (1A, 1B, 1C)
Conc 1	2 ml of cells +4 μl of Conc 1	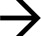 0.5 ml for well (2A, 2B, 2C)
Conc 2	2 ml of cells +4 μl of Conc 2	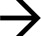 0.5 ml for well (3A, 3B, 3C)
Conc 3	2 ml of cells +4 μl of Conc 3	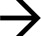 0.5 ml for well (4A, 4B, 4C)
Conc 4	2 ml of cells +4 μl of Conc 4	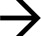 0.5 ml for well (5A, 5B, 5C)
Conc 5	2 ml of cells +4 μl of Conc 5	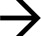 0.5 ml for well (6A, 6B, 6C)

**TABLE 4:** Plate configuration for cell viability assay.

	1	2	3	4	5	6
A	Ctrl	Conc 1	Conc 2	Conc 3	Conc 4	Conc 5
B	Ctrl	Conc 1	Conc 2	Conc 3	Conc 4	Conc 5
C	Ctrl	Conc 1	Conc 2	Conc 3	Conc 4	Conc 5
D						Unst

**TABLE 5:** Example of tubes setup.

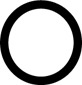 1 A	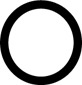 2 A	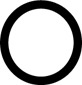 3 A	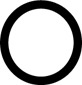 4 A	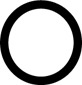 5 A	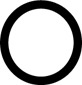 6 A	
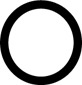 1B	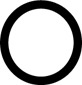 2B	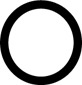 3B	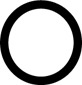 4B	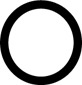 5B	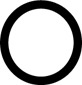 6B	
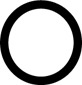 1C	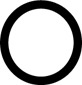 2C	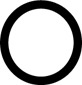 3C	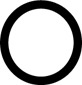 4C	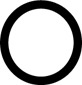 5C	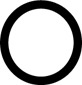 6C	
						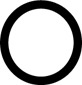 6D
THP-1 + PBS or DMSO	THP-1 + Conc 1	THP-1 + Conc 2	THP-1 + Conc 3	THP-1 + Conc 4	THP-1 + Conc 5	THP-1

Important: it is necessary to keep the tubes on ice throughout the whole process.

Cell viability is calculated by setting the gate to approximately 99% on the cells treated with the control vehicle (PBS or DMSO) and comparing the treated cells to them. The CV75 value is used to determine the concentration of test chemicals to be used in the next experiments.

**FIGURE 2:**
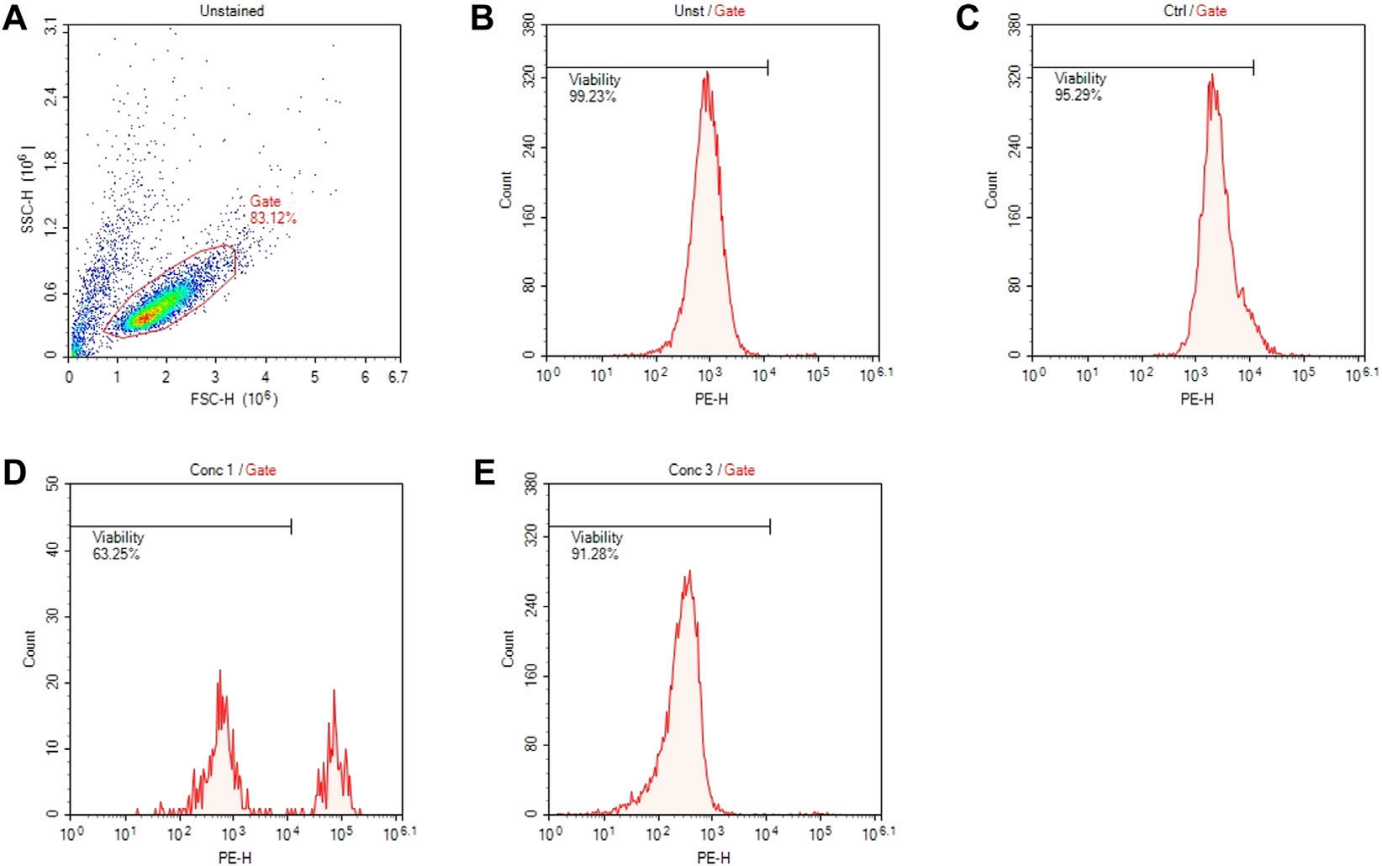
Gating strategies for cell viability assessment. **(A)** Representative dot plot diagram of forward-scatter (FSC) and side-scatter (SSC) light. The threshold level for FSC-H was set to exclude signals from cellular debris. **(B)** Representative histogram of viability of non-treated and non-stained cells (Unst). **(C)** Representative histogram of control cells stained with PI. **(D)** Representative histogram of cells treated with a concentration of drug that resulted cytotoxic (Conc 1). **(E)** Representative histogram of cells treated with a concentration of drug that resulted non-cytotoxic (Conc 3).

An example of gate setting is shown in (Figure 2).

### 3.4 Cell Treatment for the Assessment of Surface Marker Expressions (CD54 and CD86) and Release of IL-8.

Three independent experiments are performed to determine the expression of surface markers and the release of IL-8. It is incubated for different time intervals, following the scheme protocol: 24 h for the expression of CD86 and the release of IL-8. If there is no increase in the CD86 expression and/or production of IL-8, evaluation of IL-8 mRNA is continued at 3 h. If not, the cells are treated for 48 h, and the CD86/CD54 expression is analyzed.

Day 1:
  1. *Cell count:* same as previously described.
  2. Cells are brought to 10^6^ cells/ml. 500 μl of the treated cell suspension is seeded on a 24-well flat-bottom plate. The CV75 will be the maximum concentration tested. If no CV75 could be calculated, 2 mg/ml will be the maximum concentration tested.
  3. Fresh stock solutions are prepared for each experiment. A 1.5 ml micro test tube containing the 500X of the CV75 concentration (Conc 1) is prepared, and from this, three 1:2 dilutions (Conc 2, Conc 3, and Conc 4) are prepared. PBS or DMSO is used as the vehicle control (see treatment example in Table 6).
  4. Then, 500 μl of the treatment solution is plated in each well, and in one well plate, 500 μl of THP-1 without any treatment (Unst) (see plate configuration in Table 7) is plated. The cells are incubated for 24 and 48 h (depending on the marker/cytokine to be analyzed) at 37°C, 5% CO_2_, and 95% humidity. For all conditions, four wells are plated: three for the analysis of the marker, which will be analyzed in triplicates, and one for the analysis of the isotype.
Days 2 and 3:
  1. After 24 and 48 h, the tubes are prepared for flow cytometric analysis, and only for day 2, 1.5 ml micro test tubes are prepared for the collection of supernatants for IL-8 measurement, following the diagram as follows (Table 8).
  2. The plate containing the treatment is checked under an optical microscope.
  3. It is mixed well, and 450 μl of each well is taken and transferred in the respective tube.
  4. It is centrifuged at 1,200 rpm for 5′ at 4°C.
  5. Day 2: the supernatants are collected in the correspondent 1.5 ml micro test tubes, being careful not to lose the pellet, and the supernatants are conserved at −20°C.
  Day 3: the supernatants are deleted.
  6. 200 μl of the antibody solution is added for the marker or isotype solutions to the pellets. The marker solution is composed by 200 μl of PBS, and the antibody binding the marker (CD86, CD54, or HLA-DR) is diluted, following the manufacturer’s data sheet. The same must be performed for the isotype. If the antibodies are linked to different fluorophores, they can be added to the same tube. For THP-1 naïve (Unst tube), only 200 μl of PBS is added.
  7. It is incubated at 4°C for 30’.
  8. After the incubation, all the tubes are centrifuged at 1,200 rpm for 5′ at 4°C, supernatants are eliminated, washing with 200 μl of PBS, centrifuged again, eliminated supernatants, and cells are resuspended in 500 μl of PBS. The expression levels of the marker are analyzed using a flow cytometer.

**TABLE 6:** Treatment example.

Ctrl	2.5 ml of cells +5 μl PBS or DMSO	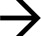 0.5 ml for well (1A, 1B, 1C, 1D)
Conc 1	2.5 ml of cells +5 μl of Conc 1	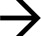 0.5 ml for well (2A, 2B, 2C, 2D)
Conc 2	2.5 ml of cells +5 μl of Conc 2	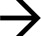 0.5 ml for well (3A, 3B, 3C, 3D)
Conc 3	2.5 ml of cells +5 μl of Conc 3	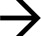 0.5 ml for well (4A, 4B, 4C, 4D)
Conc 4	2.5 ml of cells +5 μl of Conc 4	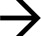 0.5 ml for well (5A, 5B, 5C, 5D)

**TABLE 7:** Plate configuration for treatment.

	1	2	3	4	5	6
A	Ctrl	Conc 1	Conc 2	Conc 3	Conc 4	
B	Ctrl	Conc 1	Conc 2	Conc 3	Conc 4	
C	Ctrl	Conc 1	Conc 2	Conc 3	Conc 4	
D	Ctrl	Conc 1	Conc 2	Conc 3	Conc 4	Unst

**TABLE 8:** Example of tubes setup.

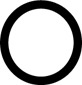 1 A	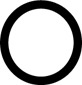 2 A	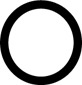 3 A	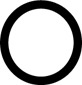 4 A	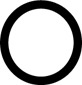 5 A		Marker
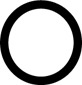 1B	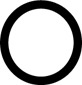 2B	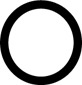 3B	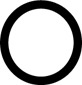 4B	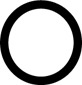 5B		Marker
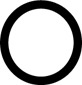 1C	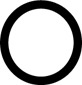 2C	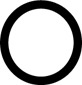 3C	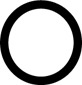 4C	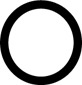 5C		Marker
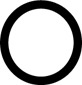 1D	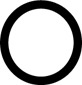 2D	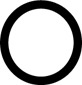 3D	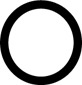 4D	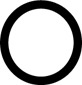 5D		Isotype
					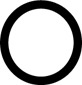 6D	
THP-1 + PBS or DMSO	THP-1 + Conc 1	THP-1 + Conc 2	THP-1 + Conc 3	THP-1 + Conc 4	THP-1	

Important: it is necessary to keep the tubes on ice throughout the whole process.

#### 3.4.1 FACS Analysis

**FIGURE 3:**
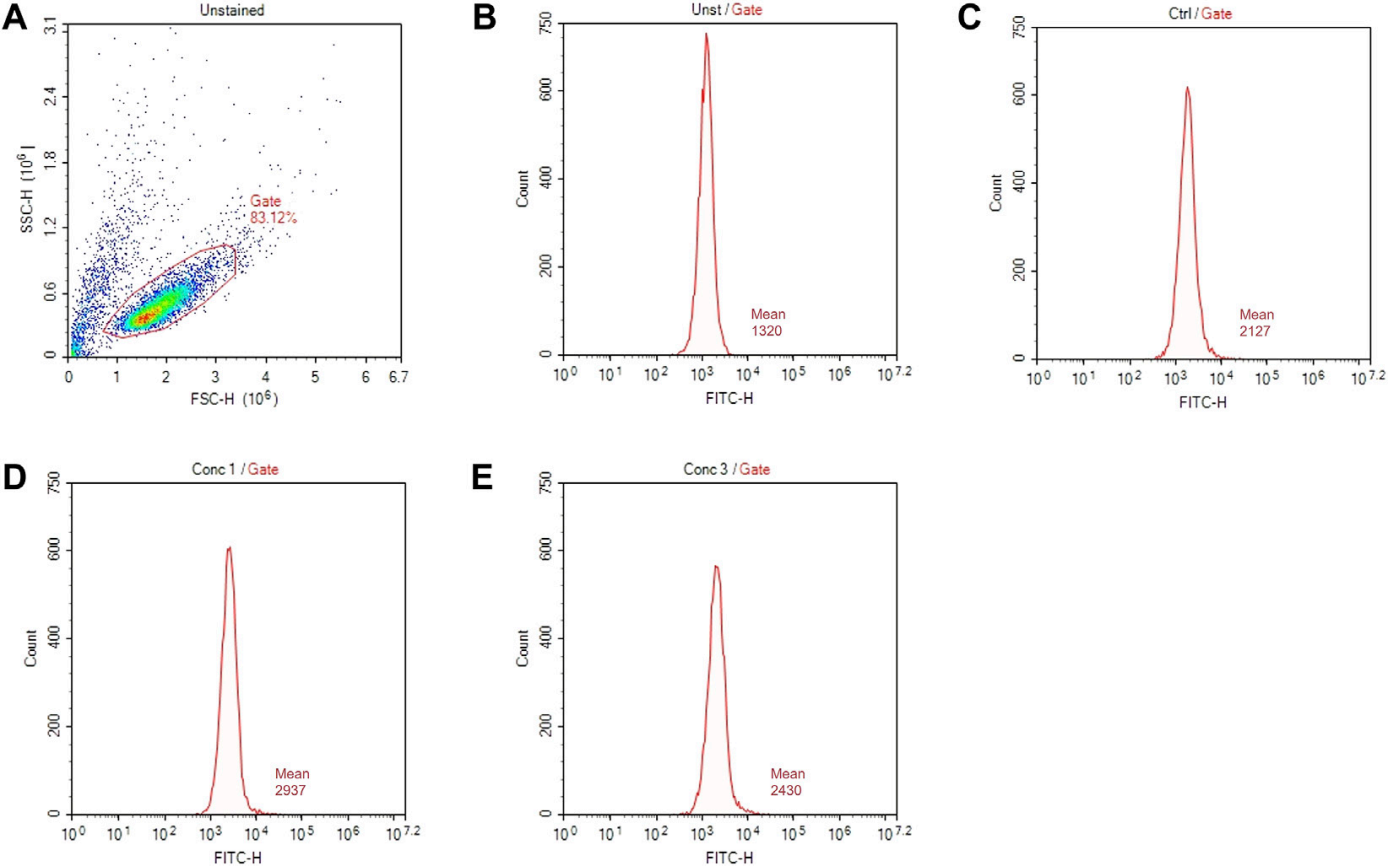
Gating strategies for marker assessment. **(A)** Representative dot plot diagram of forward-scatter (FSC) and side-scatter (SSC) light. The threshold level for FSC-H was set to exclude signals from cellular debris. **(B)** Representative histogram of marker expression of non-treated and non-stained cells (Unst). **(C)** Representative histogram of the marker expression of control cells **(D)** Representative histogram of cells treated with a higher non-cytotoxic concentration of drugs (Conc 1). **(E)** Representative histogram of cells treated with a middle non-cytotoxic concentration of drugs (Conc 3).

The surface marker expression is analyzed by flow cytometry with the acquisition in channels FL-1 (FITC) and FL-3 (PE). A total of 10,000 events are acquired. An example of gate setting is shown in (Figure 3).

Based on the geometric mean fluorescence intensity (MFI), the relative fluorescence intensity (RFI) of markers for vehicle control cells and chemical-treated cells are calculated according to the following equation.

$$RFI=\frac{\text{MFI of chemical treated cells}-\text{MFI of chemical treated isotype control cells }}{\text{MFI of vehicle treated control cells}-\text{MFI of vehicle treated isotype control cells}}.$$

### 3.5 Determination of the IL-8 Release by ELISA.

Day 1:

1. Wells are coated with the capture antibody
  1.1. The lyophilized capture antibody (anti-human IL-8 Capture-Antibody) is reconstituted with 500 μl PBS.
  1.2. The capture-antibody 1:100 is diluted in a coating buffer (e.g., PBS).
  1.3. 100 μl of the diluted IL-8 capture-antibody is pipetted in each well.
  1.4. The 96-well plate is sealed and incubated O.N. at room temperature.

Day 2:

2. The capture antibody is removed completely washing 3 times with a washing buffer (e.g., PBS +0.05% Tween 20). For the last wash, any residual buffer is removed by tapping on a paper towel.
3. The plate is blocked with the blocking buffer
  3.1. Blocking buffer is prepared (e.g., PBS +2% BSA).
  3.2. In each well, 200 μl of blocking buffer is pipetted. The plate is sealed and incubated for 1 h at room temperature.
4. The blocking buffer is removed completely washing three times with the washing buffer. For the last wash, any residual buffer is removed by tapping on a paper towel.
5. Addition of standard and samples
  5.1 Standards are prepared, and eventually, the samples are diluted during incubation with the blocking buffer
  5.2. Standard: serial dilutions are prepared in reagent diluent (e.g., PBS +2% BSA) and is begun with a high standard and end with blanks. The standard vial of the Immunotools human IL-8 ELISA set contains 50 ng of lyophilized rhIL-8. This is reconstituted in exactly 1 ml reagent diluent (stock solution = 50 ng/ml and range curve 8—500 pg/ml). In the following table (Table 9), an example of the serial dilutions of the recombinant human IL-8 antibody required for standard IL-8 curve is given.
  5.3. Samples are diluted in such a way that their OD values will be in the linear part of the standard curve.
  5.4. 100 μl of standards are pipetted from 0 pg/ml (dilution buffer) to 500 pg/ml and samples per well.
  5.5. The plate is sealed and incubated for 2 h at room temperature.
6. The standard and samples are removed and washed five times with the washing buffer. For the last wash, any residual buffer is removed by tapping on a paper towel.
7. Addition of the biotinylated detector-antibody
  7.1. The lyophilized detector-antibody is reconstituted in 500 μl blocking buffer. The detector-antibody is diluted at 1:100 in the reagent diluent.
  7.2. 100 μl of the detection antibody is pipetted for each well; the plate is sealed and incubated for 2 h at room temperature.
8. The detection antibody is removed and washed five times with the washing buffer. For the last wash, any residual buffer is removed by tapping on a paper towel.
9. Addition of Poly-HRP-Streptavidin
  9.1 Poly-HRP-Streptavidin 1:1,000 is diluted in the reagent diluent.
  9.2 100 μl of streptavidin is pipetted in each well; the plate is sealed and incubated 30′ at room temperature.
  The solution of TMB is warmed to room temperature before use.
10. Poly-HRP-Streptavidin is removed and washed five times with the washing buffer. For the last wash, any residual buffer is removed by tapping on a paper towel.
11 TMB substrate solution is added.
  11.1 In each well, 100 μl of the TMB substrate solution is pipetted.
  11.2. The plate (without a plate sealer) is incubated ∼8–20 min at room temperature in the dark (covered with an aluminum foil). When the enzymatic color reaction is sufficient, the experiment is proceeded, and the plate is read, without stopping the reaction, at 595 nm. In an alternative, 50 μl of the stop solution (e.g., 2 M H_2_SO_4_) is added to each well. The absorbance is read at 450 nm.

**TABLE 9:** Dilution of the recombinant human IL-8 antibody required for a standard curve.

Final conc. IL-8	X-μl of rec IL-8 standard at 50 ng/ml	X-μl of previous dilution	X-μl of dilution buffer
500 pg/ml	2 μl		198
250 pg/ml	-	100 μl of the 500	100
125 pg/ml	-	100 μl of the 250	100
62.5 pg/ml	-	100 μl of the 125	100
31.25 pg/ml	-	100 μl of the 61.5	100
15,625 pg/ml	-	100 μl of the 31.25	100
7,813 pg/ml	-	100 μl of the 15,625	100

From the raw data (measured absorbance values at a wavelength of 595 or 450 nm), the mean absorbance is calculated for each set of standards and samples. *R* ^2^ of the standard curve should be >0.900. OD values of the samples should be within the linear part of the standard curve. When samples do not meet these criteria, they should be re-tested, diluting the sample accordingly. During the ELISA, the vehicle and positive control are also re-tested, if present. Results are expressed as pg/ml.

### 3.6 Prediction Model

Each drug is tested in three independent experiments to derive a single prediction (a sensitizer or non-sensitizer). Each experiment must be performed on different days. Results are expressed as the stimulation index (SI). Data are reported as mean ± SEM, and statistical analysis is performed by analysis of variance (ANOVA), followed by Dunnett’s multiple comparison test. Effects are designed significant if *p* ≤ 0.05. If the RFI of CD86 at 24 h is ≥ 1.5 at any tested dose (with ≥75% of cell viability) and/or the release of IL-8 is statistically significant, in at least two out of three independent experiments, for at least one of the concentrations tested, the drug is considered as a sensitizer. If not, the analyses continued, and if the expression of IL-8 mRNA at 3 h is ≥3.0, the drug is considered as a sensitizer. If not, the analyses continued, and if the RFI of CD86 and/or CD54 at 48 h is ≥ 1.5 at the tested dose (with ≥75% of cell viability), in at least two out of three independent experiments, for at least one of the concentrations tested, the drug is considered as a sensitizer. If not, the drug is considered definitely as a non-sensitizer for this method.

### 3.7 Cell Treatment for IL-8 mRNA Expression Assessment

Three independent experiments are performed to determine the expression of IL-8 mRNA.

Day 1:
*1. Cell count:* as previously described.
  2. The cells are brought to 10^6^ cells/ml. 3 ml of the cell suspension is seeded on a 6-well flat-bottom plate and treated with increasing non-cytotoxic drug concentrations (previously found).
  3. Fresh stock solutions are prepared for each experiment. A 1.5 ml micro test tube containing the 500X of the CV75 concentration (Conc 1) is prepared, and from this, three 1:2 dilutions are prepared to obtain the lower concentrations. The treatment solutions are prepared by adding the working solutions to the cells. PBS or DMSO is used as the vehicle control (see treatment example in Table 10).
  4. Then, 3 ml of the treatment solution is added in each well (see plate configuration in Table 11).
After 3 h:
  1. After 3 h of incubation, the tubes are prepared for mRNA extraction.
  2. The plate containing the treatment is checked under an optical microscope.
  3. It is mixed well, and all the contents (almost 3 ml) are transferred in the respective tubes.
  4. It is centrifuged at 1,200 rpm for 5′ at 25°C.
  5. The supernatant is discarded.
  6. 2 ml of sterile PBS is added in each tube, and the cells are resuspended.
  7. It is centrifuged at 1,200 rpm for 5′ at 25°C.
  8. The supernatant is discarded, and the tubes are stored on ice.
  9. Under a chemical fume cabinet, 1,000 μl of TRI reagent is added for the lysis of the cells. After the addition of the reagent, the cell lysate should be passed several times through a pipette to form a homogenous lysate.
  10. Each tube is vortexed for 1′, and all samples are transferred in sterile 1.5 ml micro test tubes. To ensure the complete dissociation of nucleoprotein complexes, the sample is allowed to stand for 5′ at room temperature.
  11. 200 μl of chloroform is added slowly along the wall of the 1.5 ml micro test tube (observe the separation between the pink TRI reagent and chloroform).
  12. Each 1.5 ml micro test tube is vortexed for 1’ (the solution became opaque), and the sample is allowed to stand for 5–10′ at room temperature.
  13. It is centrifuged at 12,000 x *g* for 15′ at 4°C.
  14. Three different layers will be obtained: the aqueous phase containing RNA is taken and transferred in a new sterile 1.5 ml micro test tube.
  15. 500 μl of isopropanol is added in each test tube and vortexed for 1′ each.
  16. The samples are stored at −20°C for 24 h (optional—1 h).
Day 2:
RNA Extraction and Quantification.
  1. If stored at −20°C, the samples are thawed and vortexed for 1′ each.
  2. It is centrifuged at 12,000 x *g* for 15′ at 4 °C.
  3. The supernatant is discarded, and 700 μl of ethanol 75% is added.
  4. The samples are vortexed for 1′ each.
  5. It is centrifuged at 7,500 x *g* for 5′ at 4°C.
  6. The supernatant is discarded.
  7. The RNA pellet is dried for 30′ under a vacuum. The RNA pellet is not allowed to dry completely as this will greatly decrease its solubility.
  8. 20 μl of nuclease-free water is added to the RNA pellet. To facilitate dissolution, it is mixed by repeated pipetting with different micropipettes and is gently vortexed.
  9. The sample is transferred to the thermoblock at 60°C for 10’.
  10. All samples are vortexed briefly.
  11. The RNA concentration is read with a Nanodrop. Final preparation of RNA should have the A_260_/A_280_ ratio ≥1.7 and not over 2. The concentration of RNA is calculated in each sample.

**TABLE 10:** Treatment example.

Ctrl	3.5 ml of cells +7 μl PBS or DMSO	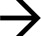 3 ml in well 1 A
Conc 1	3.5 ml of cells +7 μl of Conc 1	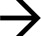 3 ml in well 2 A
Conc 2	3.5 ml of cells +7 μl of Conc 2	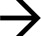 3 ml in well 3 A
Conc 3	3.5 ml of cells +7 μl of Conc 3	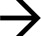 3 ml in well 1B
Conc 4	3.5 ml of cells +7 μl of Conc 4	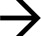 3 ml in well 2B

**TABLE 11:** Plate configuration for treatment.

	1	2	3
A	Ctrl	Conc 1	Conc 2
B	Conc 3	Conc 4	

Reverse Transcription

The following steps are taken from the official protocol QuantiNova Reverse Transcription Kit by QIAGEN. If using different kits, it is advised to follow manufacturer’s instructions.

1. The RNA is kept on ice. Genomic DNA (gDNA) removal mix and reverse transcription enzyme are thawed on ice and transcription mix and RNase-free water at 25°C. All the thawed components are briefly centrifuged and kept on ice.
2. The RNA volume is calculated to have a final concentration of 5 μg.
3. The genomic DNA removal reaction is prepared on ice, as explained below (for one sample):
  - gDNA removal mix: 2 μl
  - Template RNA: Variable
  - RNase-free water: Variable

Total volume: 15 μl

4. It is mixed and then kept on ice.
5. It is incubated for 2′ at 45°C, using the thermocycler, and subsequently placed on ice.
6. The reverse transcription master mix is prepared on ice, as explained below (for one sample):
  - Reverse transcription enzyme: 1 μl
  - Reverse transcription mix: 4 μl
  - Template RNA following gDNA removal (step 5): 15 μl

Total volume: 20 μl

7. It is mixed and then kept on ice.
8. It is incubated for 3′ at 25°C (annealing), 10′ at 45°C (reverse-transcription), and 5′ at 85°C (inactivation) using the thermocycler.
9. The reverse transcription reaction 1:10 is diluted in nuclease-free water on ice; if proceeded directly with real-time PCR, or for long-term storage, the reverse transcription reaction is kept at –20 °C.
Day 3:

Real-Time PCR.

1. 2x QuantiNova SYBR Green PCR Master Mix, QuantiNova Yellow Template Dilution Buffer, template cDNA, primers, and RNase-free water are thawed. They are mixed and kept on ice.
2. The real-time PCR Mix is prepared on ice accordingly, as explained later (for one sample):
  - 2x QuantiNova SYBR Green PCR Master Mix: 10 μl
  - Primer: 2 μl
  - RNase-free water: 6 μl
  - cDNA: 2 μl

Total volume: 20 μl

3. 2 μl of cDNA in each well of the PCR plate and 18 μl of the Mix are mixed and dispensed, with the appropriate primer (CXCL8_1) or control (RRN18S_1).
4. It is incubated for 2′ at 95°C (initial activation) and subsequently 40 cycles of 5″ at 95°C (denaturation) and 10″ at 60°C (combined annealing-extension), using the thermocycler.
5. The quantification of the transcripts is performed the by 2^−ΔΔCT^ method.

### 3.8 Time Considerations

**FIGURE 4:**
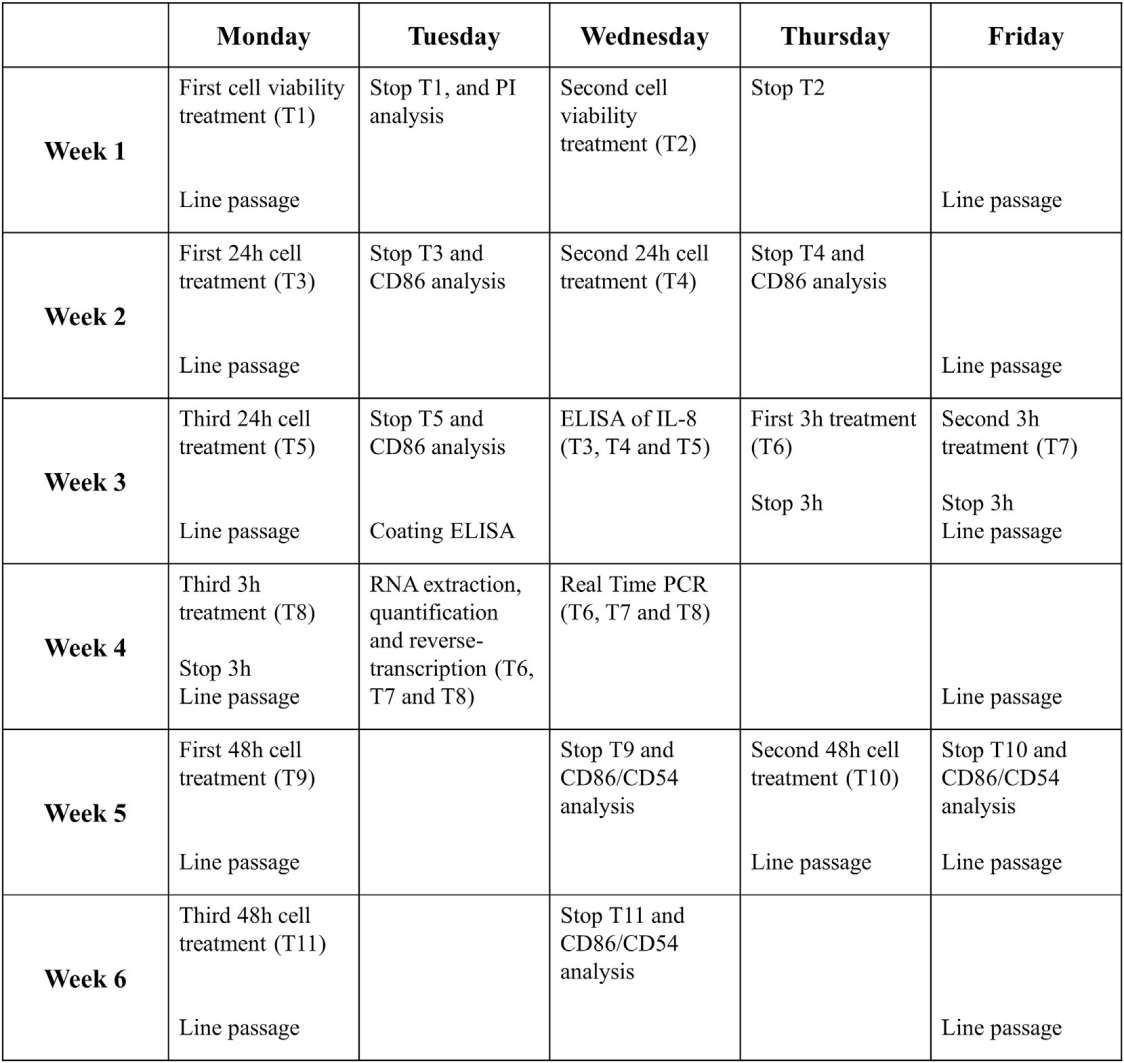
Timeline of one complete drug analysis. The schedule represents the time required for the analysis of a drug which resulted to be non-sensitizer, for which all the steps of the protocol are required. The times can be reduced if the drug proves the sensitizing potential already at earlier stages of the protocol.

6 weeks are needed for a complete analysis of a drug following this protocol. Figure 4 shows an example of the job setting more in detail:

- The first week will be necessary to evaluate the cell viability and decide the range of non-cytotoxic concentrations to be used for subsequent experiments (T1 and T2).
- Then, in the second and third week, the expression of CD86 and the release of IL-8 after 24 h of treatment will be analyzed (T3, T4, and T5). If the RFI of CD86 at 24 h ≥ 1.5 at any tested dose (with cell viability ≥75%) and/or the release of IL-8 is statistically significantly upregulated, in at least two out of three independent experiments, for at least one of the concentrations tested, the drug is considered a sensitizer.
- If not, the analyses will continue, and in the third and fourth week, the expression of IL-8 mRNA after 3 h of treatment will be analyzed (T6, T7, and T8). If the IL-8 mRNA expression at 3 h ≥ 3.0, the drug is considered a sensitizer.
- If not, the analyses will continue, and during the fifth and sixth week, the expressions of CD86 and CD54 after 48 h of treatment will be analyzed (T9, T10, and T11). If the RFI of CD86 and/or CD56 at 48 h ≥ 1.5 at any tested dose (with cell viability ≥75%), in at least two out of three independent experiments, for at least one of the concentrations tested, the drug is considered a sensitizer. If not, the drug is definitely considered a non-sensitizer for this method.

The timetable proposed take into consideration the “worst case” that is represented by the failure in any response induced by the tested drugs. In this case, chemicals tested are considered as non-sensitizers. Obviously, the timeline become shorter if the activation of the selected markers is acquired in the first steps. To set-up the THP-1 activation assay, sensitizer drugs reported in the literature were used. Overall, data obtained support the main classification reported in the literature for each drugs tested.

## 4 Results and Discussion

DHRs are common among drugs (6–15% of all adverse drug reactions), cause severe patient discomfort, and in some cases, can lead to life-threatening situations. Despite this serious problem and the negative economic impact deriving from market withdrawal of such drugs and high hospitalization costs, nowadays, there are no standard validated methods *in vitro* or *in vivo* to evaluate the sensitizing potential of drugs in the preclinical phase. The use of this *in vitro* NAM, also considering the results already present in the literature (Corti et al., 2015; Iulini et al., 2020), could fill this gap.

Published results (Corti et al., 2015; Iulini et al., 2020) shown that all drugs tested, for which DHRs are known, were able to modulate the phenomena underlying sensitization, inter alia CD54, CD86, and IL-8 expressions. These results are summarized and shown in Supplementary Material 1. The modified THP-1 activation assay allowed the correct identification of all the tested drugs as sensitizers and as non-sensitizers for the negative ones. The modified THP-1 activation is a valid model that should be incorporated into drug development in the preclinical phase.

Drugs with different structures, mechanism of action, and therapeutic application have been tested, supporting the evidence that this method can be used for different types of drugs regardless of their molecular forms. The concentrations at which the drugs were tested were obtained starting from the highest concentration that did not confer cytotoxicity to THP-1 cells (viability in cells greater than 75%) by the PI assay. Clonidine, ofloxacin, procainamide, and streptozotocin are drugs associated with a relatively high incidence of immune-mediated hypersensitivity reactions (Corti et al., 2015). They led to an upregulation of the selected markers (CD86 and IL-8) at 24h, while sulfamethoxazole requires 48 h of treatment. Methyl salicylate and probenecid, which can cause irritant or allergic contact dermatitis and anaphylactic reactions (Corti et al., 2015), were also able to induce the upregulation of the selected markers. Furthermore, drugs associated with DHRs, for which a correlation with specific HLA was established (Iulini et al., 2020), namely, abacavir, carbamazepine, and allopurinol, induced a higher upregulation of CD86 at 24 h, while clozapine and flucloxacillin needed 48 h for the upregulation of CD54. For these latter drugs, the ability to upregulate HLA-DR, as an indicator of the ability to further activate DCs, was also investigated. Finally, metformin, selected as the negative control as there is no evidence of its possible action at the immune level, did not induce any significant changes in the CD86, CD54 expression, or IL-8 release both at 24 and 48 h. Furthermore, it also failed in the induction of IL-8 mRNA.

As anticipated by Iulini et al. (2020), the predictive capacity of the method has also been investigated toward drugs associated with DHRs, for which is present in the literature a correlation with specific HLAs. The evolution of pharmacogenetics showed a correlation between HLAs and systemic hypersensitivity reactions, indicating the HLA as one of the determining factors linking drug exposure and unwanted immune responses. However, not all patients expressing specific HLA risk genotypes are sensitive, and many others without risk alleles could also develop drug hypersensitivity reactions, suggesting that several other different factors are likely to be involved in the development of DHRs. As data presented in Supplementary Material S1 shown, only abacavir and flucloxacillin were able to upregulate HLA-DR. This biomarker is the major histocompatibility complex class II cell surface receptor that presents peptides deriving from extracellular proteins. Studies have shown (Illing et al., 2016) that abacavir and flucloxacillin can activate T cells mainly through the hapten concept mechanism, which involves antigen processing to form a peptide–antigen complex, thus explaining why abacavir and flucloxacillin were able to induce the MHC class II expression. On the contrary, literature data support that carbamazepine and allopurinol binds directly on the antigen-binding groove of the HLA molecule (p-i mechanism), and thus, there is no need for haptenization of the drug to activate T cells (Illing et al., 2016). As a result, this could indicate why carbamazepine and allopurinol were not able to induce the expression of HLA-DR.

The originally developed THP-1 assay based on the release of IL-8 alone has been modified to identify sensitizing or non-sensitizing drugs. The result showed that the exposure of THP-1 cells to sensitizing drugs resulted, in most cases, in a dose-related higher release of IL-8 and upregulation of the CD86 expression, with some differences among drugs, markers, and times of exposure. The use of a single marker to identify the sensitizing potential of drugs would lead to inadequate recognition of them. In fact, there is no marker able to discriminate more significantly than another: some drugs are more sensitive to IL-8, others to CD86 or CD54. Indeed, different drugs were able to activate DCs through the modulation of different markers and following different kinetics, and we can speculate that it may be correlated with their sensitizing potency. For example, if considering the release of IL-8 at 24 h alone, it would lead to the categorization as non-sensitizing drugs sulfamethoxazole and clozapine, for which 48 h of treatment was required. The combination of CD86 and CD54 expressions at 48 h will allow to correctly identify more drugs tested. The use of the IL-8 mRNA expression at 3 h can offer an alternative and increase the confidence in the negativity of a drug. The choice to add IL-8 mRNA expression investigation is based on a previous observation made on chemical allergens that fail to induce the IL-8 release at either 24 or 48 h but able to induce the IL-8 mRNA expression after 3 h of treatment (Galbiati et al., 2012). Therefore, the assessment of the combination of these markers is necessary for a more adequate classification of the sensitizing potential of drugs.

The THP-1 activation assay represents a protocol that can be used for the correct identification of drugs for which there are pieces of *in vivo* evidence of DHRs. As previously mentioned, the protocol takes into consideration only one of the key events of AOP for sensitization (Key event 3). Of course, a possible limitation is that this protocol considers only one of the mechanisms involved in drug hypersensitivity, missing other factors and mechanisms, previously described in the Introduction. The choice of this key event was based on a successful outcome of the proposed model, high reproducibility, and an easy and rapid use of the protocol as first screening for the activation of the immune system during the development of drugs in the preclinical phase. After the collection of the data obtained with the protocol, it is necessary to continue the investigation of the sensitizing potential in the subsequent phases of the development of the pharmacological entity. Therefore, a battery of methods (the integrated testing strategy) is needed, such as the combination with the investigation of other key events underneath immune activation.

A limitation of the proposed protocol is that drugs are tested as they are, in their original form, and not with their active metabolites. Frequently, the hypersensitivity reactions to drugs can be caused by metabolites generated by drug biotransformation and not by the parent drug (e.g., procainamide and streptozotocin). Nevertheless, also in absence of hepatic biotransformation in the proposed model, the method was able to positively classify parental drugs as sensitizers. Therefore, the proposed protocol allows to classify the original drug as a sensitizer or non-sensitizer; as a first screening, that should be followed by more in-depth investigations, also regarding their metabolite activities.

The THP-1 activation assay is proposed as a protocol to be integrated during the sensitization studies conducted on a new pharmacological entity to predict the drug potential to induce DHRs. This, in a broad vision, helps to reduce and replace animal testing. The data obtained in our laboratory and presented in Corti et al. (2015) and Iulini et al. (2020) indicate that through the protocol, it was possible to correctly identify drugs for which there were *in vivo* evidence of DHRs.

## Data Availability

The original contributions presented in the study are included in the article/Supplementary Material, further inquiries can be directed to the corresponding author.
