# Supplementary material for: The Modified THP-1 Activation Assay for the In Vitro Identification of Drug-Inducing Systemic Hypersensitivity
Source: Front Toxicol. 2022 Mar 3;4:814050. doi: 10.3389/ftox.2022.814050 (PMC8915845; doi:10.3389/ftox.2022.814050)
Supplement: Supplementary file 1 [file Table1.DOCX]

# Supplementary materials

| **Drug** | **CAS#** | **Literature evidence DHR** | **THP-1 activation assay** | | | | | **Protocol**  **prediction** |
| --- | --- | --- | --- | --- | --- | --- | --- | --- |
|  |  |  | CD86 24h | IL-8 24h | CD86 48h | CD54 48h | IL-8 3h |  |
| **Abacavir sulphate** | 188062-50-2 | Associated with HLA-B*57:01 ^[1]^ | ↑ | - | NA | NA | NA | Sensitizer ^[b]^ |
| **Allopurinol** | 315-30-0 | Associated with HLA-B*58:01 ^[2]^ | ↑ | ↑ | NA | NA | NA | Sensitizer ^[c]^ |
| **Carbamazepine** | 298-46-4 | Associated with HLA-B*15:02 and HLA-A*31:01 ^[3]^ | ↑ | - | NA | NA | NA | Sensitizer ^[b]^ |
| **Clonidine** | 4205-91-8 | High incidence ^[4]^ | ↑ | ↑ | NA | NA | NA | Sensitizer ^[a]^ |
| **Clozapine base** | 5786-21-0 | Associated with HLA-B*59:01 and HLA-DQB1*05:02 ^[5]^ | - | ↑ | - | ↑ | NA | Sensitizer ^[b]^ |
| **Flucloxacillin** | 1847-24-1 | Associated with HLA-B*57:01 ^[6]^ | ↑ | ↑ | ↑ | ↑ | NA | Sensitizer ^[c]^ |
| **Metformin hydrochloride** | 1115-70-4 | No evidence ^[4]^ | - | - | - | - | - | Non sensitizer ^[a]^ |
| **Methyl salicylate** | 119-36-8 | Irritant or allergic contact dermatitis and anaphylactic reactions ^[7]^ | ↑ | ↑ | NA | NA | NA | Sensitizer ^[a]^ |
| **Ofloxacin** | 82419-36-1 | High incidence ^[8]^ | ↑ | ↑ | NA | NA | NA | Sensitizer ^[a]^ |
| **Probenecid** | 56-66-9 | Irritant or allergic contact dermatitis and anaphylactic reactions ^[9]^ | - | ↑ | ↑ | NA | NA | Sensitizer ^[a]^ |
| **Procainamide** | 614-39-1 | High incidence ^[10]^ | ↑ | ↑ | NA | NA | NA | Sensitizer ^[a]^ |
| **Streptozotocin** | 18883-66-4 | Associated with HLA-DQB1*0601 ^[11]^ and cutaneous reactions ^[12]^ | ↑ | ↑ | NA | NA | NA | Sensitizer ^[a]^ |
| **Sulfamethoxazole** ^[a]^ | 723-46-6 | High incidence ^[13]^ | - | ↑ | ↑ | NA | NA | Sensitizer |

**Legend:** ↑ : statistical significant up-regulation; - : no statistical significant evidence; NA : not assessed; [a] results published in Corti et al. (2015); [b] results published in Iulini et al. (2020); [c] results not published yet.

**Table 1: Summary of the data obtained by Corti et al. (2015) and Iulini et al. (2020) using the THP-1 activation assay.** For all drug is reported: CAS number, literature evidence of induce DHRs, results obtained with the THP-1 activation assay and prediction of sensitizer or non sensitizer potential of drug for the protocol use. For each drug analyzed, the protocol confirmed the data present in the literature.

1. Mounzer, K., Hsu, R., Fusco, J. S., Brunet, L., Henegar, C. E., Vannappagari, V., Stainsby, C. M., Shaefer, M. S., Ragone, L., & Fusco, G. P. (2019). HLA-B*57:01 screening and hypersensitivity reaction to abacavir between 1999 and 2016 in the OPERA® observational database: a cohort study. *AIDS research and therapy*, 16(1), 1. https://doi.org/10.1186/s12981-019-0217-3
2. Hung, S. I., Chung, W. H., Liou, L. B., Chu, C. C., Lin, M., Huang, H. P., Lin, Y. L., Lan, J. L., Yang, L. C., Hong, H. S., Chen, M. J., Lai, P. C., Wu, M. S., Chu, C. Y., Wang, K. H., Chen, C. H., Fann, C. S., Wu, J. Y., & Chen, Y. T. (2005). HLA-B*5801 allele as a genetic marker for severe cutaneous adverse reactions caused by allopurinol. *Proceedings of the National Academy of Sciences of the United States of America*, 102(11), 4134–4139. https://doi.org/10.1073/pnas.0409500102
3. Khor, A. H., Lim, K. S., Tan, C. T., Kwan, Z., Tan, W. C., Wu, D. B., & Ng, C. C. (2017). HLA-A*31: 01 and HLA-B*15:02 association with Stevens-Johnson syndrome and toxic epidermal necrolysis to carbamazepine in a multiethnic Malaysian population. *Pharmacogenetics and genomics*, 27(7), 275–278. https://doi.org/10.1097/FPC.0000000000000287
4. Weaver, J. L., Chapdelaine, J. M., Descotes, J., Germolec, D., Holsapple, M., House, R., Lebrec, H., Meade, J., Pieters, R., Hastings, K. L., & Dean, J. H. (2005). Evaluation of a lymph node proliferation assay for its ability to detect pharmaceuticals with potential to cause immune-mediated drug reactions. *Journal of immunotoxicology*, 2(1), 11–20. https://doi.org/10.1080/15476910590930100
5. Goldstein, J. I., Jarskog, L. F., Hilliard, C., Alfirevic, A., Duncan, L., Fourches, D., Huang, H., Lek, M., Neale, B. M., Ripke, S., Shianna, K., Szatkiewicz, J. P., Tropsha, A., van den Oord, E. J., Cascorbi, I., Dettling, M., Gazit, E., Goff, D. C., Holden, A. L., Kelly, D. L., … Sullivan, P. F. (2014). Clozapine-induced agranulocytosis is associated with rare HLA-DQB1 and HLA-B alleles. *Nature communications*, 5, 4757. https://doi.org/10.1038/ncomms5757
6. Daly, A. K., Donaldson, P. T., Bhatnagar, P., Shen, Y., Pe'er, I., Floratos, A., Daly, M. J., Goldstein, D. B., John, S., Nelson, M. R., Graham, J., Park, B. K., Dillon, J. F., Bernal, W., Cordell, H. J., Pirmohamed, M., Aithal, G. P., Day, C. P., DILIGEN Study, & International SAE Consortium (2009). HLA-B*5701 genotype is a major determinant of drug-induced liver injury due to flucloxacillin. *Nature genetics*, 41(7), 816–819. https://doi.org/10.1038/ng.379
7. Chan T. Y. (1996). Potential dangers from topical preparations containing methyl salicylate. *Human & experimental toxicology*, 15(9), 747–750. https://doi.org/10.1177/096032719601500905
8. Nam, Y. H., Kim, J. E., Kim, S. H., Jin, H. J., Hwang, E. K., Shin, Y. S., Ye, Y. M., & Park, H. S. (2012). Immunologic evaluation of ofloxacin hypersensitivity. *Allergy, asthma & immunology research*, 4(6), 367–369. https://doi.org/10.4168/aair.2012.4.6.367
9. Myers, K. W., Katial, R. K., & Engler, R. J. (1998). Probenecid hypersensitivity in AIDS: a case report. Annals of allergy, asthma & immunology: official publication of the American College of Allergy, *Asthma, & Immunology*, 80(5), 416–418. https://doi.org/10.1016/S1081-1206(10)62994-8
10. Kim, S. Y., & Benowitz, N. L. (1990). Poisoning due to class IA antiarrhythmic drugs. Quinidine, procainamide and disopyramide. *Drug safety*, 5(6), 393–420. https://doi.org/10.2165/00002018-199005060-00002
11. Rajagopalan, G., Kudva, Y. C., Chen, L., Wen, L., & David, C. S. (2003). Autoimmune diabetes in HLA-DR3/DQ8 transgenic mice expressing the co-stimulatory molecule B7-1 in the beta cells of islets of Langerhans. *International immunology*, 15(9), 1035–1044. https://doi.org/10.1093/intimm/dxg103
12. Weaver, J. L., Chapdelaine, J. M., Descotes, J., Germolec, D., Holsapple, M., House, R., Lebrec, H., Meade, J., Pieters, R., Hastings, K. L., & Dean, J. H. (2005). Evaluation of a lymph node proliferation assay for its ability to detect pharmaceuticals with potential to cause immune-mediated drug reactions. *Journal of immunotoxicology*, 2(1), 11–20. https://doi.org/10.1080/15476910590930100
13. Zawodniak, A., Lochmatter, P., Beeler, A., & Pichler, W. J. (2010). Cross-reactivity in drug hypersensitivity reactions to sulfasalazine and sulfamethoxazole. *International archives of allergy and immunology*, 153(2), 152–156. https://doi.org/10.1159/000312632
14. Corti, D., Galbiati, V., Gatti, N., Marinovich, M., Galli, C. L., & Corsini, E. (2015). Optimization of the THP-1 activation assay to detect pharmaceuticals with potential to cause immune mediated drug reactions. *Toxicology in vitro*, 29(7), 1339–1349. https://doi.org/10.1016/j.tiv.2015.04.012
15. Iulini, M., Maddalon, A., Galbiati, V., Marinovich, M., & Corsini, E. (2020). In vitro identification of drugs inducing systemic hypersensitivity reactions known in vivo to be associated with specific HLA genotypes. *Toxicology in vitro*, 68, 104953. https://doi.org/10.1016/j.tiv.2020.104953
